# Embolization alone is as effective as TACE for unresectable HCC: systematic review and meta-analysis of randomized controlled trails

**DOI:** 10.1186/s12876-024-03282-z

**Published:** 2024-06-07

**Authors:** Guoliang Wang, Jinxiang Zhang, Hao Liu, Qichang Zheng, Ping Sun

**Affiliations:** 1 Department of Hepatobiliary Surgery, Union Hospital, Tongji Medical College, Huazhong University of Science and Technology, Wuhan, 430022 China; 2 Department of Emergency Surgery, Union Hospital, Tongji Medical College, Huazhong University of Science and Technology, Wuhan, 430022 China; 3 Department of Surgery, University of Pittsburgh, Pittsburgh, PA 15213 USA

**Keywords:** Hepatocellular carcinoma, Transarterial chemoembolization, Transarterial embolization, Meta-analysis

## Abstract

**Background:**

Despite transarterial chemoembolization (TACE) was recommended as first line therapy for intermediate hepatocellular carcinoma (HCC), the efficacy of transarterial embolization (TAE) has not been widely recognized. This work was to determine whether TAE was as effective and safe as TACE for unresectable HCC.

**Methods:**

We performed a systematic search of electronic databases and other sources for randomized controlled studies (RCTs) comparing TAE with TACE for unresectable HCC. Results were expressed as Hazard Ratio (HR) for survival and Odds Ratio (OR) for dichotomous outcomes using RevMan 5.4.1.

**Results:**

We included 6 trials with 683 patients. The risk of bias of included RCTs was from unclear to high risk. There were no significant differences between TACE and TAE for progression-free survival (HR 0.83, 95% CI 0.45–1.55; *p* = 0.57), overall survival (HR 1.10, 95% CI 0.90–1.35; *p* = 0.36), and objective response rate (OR 1.17, 95% CI 0.80–1.71; *p* = 0.42) without obvious publication bias. Sensitivity analyses confirmed the robustness of the results. TAE group reported similar or less adverse effects than TACE group in all the studies.

**Conclusions:**

Our study demonstrated that TAE was as effective as TACE. Since TAE was simpler, cheaper and had less adverse effects than TACE, TAE should be a better choice in most cases where TACE was indicated for unresectable HCC.

**Supplementary Information:**

The online version contains supplementary material available at 10.1186/s12876-024-03282-z.

## Background

Primary liver cancer is the sixth most commonly diagnosed cancer and the third leading cause of cancer death world-wide in 2020, with hepatocellular carcinoma (HCC) accounts for 75-85% [1]. Resection, ablation and transplantation are widely accepted as radical treatment, but only appropriate for minority patients with relatively early staged tumors [2]. For patients with tumors of intermediate and advanced stage, transarterial chemoembolization (TACE) [3, 4] and systemic therapies [5, 6] are first line choices respectively.

The first case of transarterial chemotherapy was happened by accidental administration of HN_2_ in to the branchial artery of a patient with Hodgkin’s disease. Since then, transarterial infusion chemotherapy has been applied for localized tumors [7, 8]. Ecker and his associates introduced Hepatic arterial infusion chemotherapy (HAIC) by insertion of catheter through gastroduodenal artery in open surgery in 1962. The patients were tolerated well without severe systemic symptoms [9]. In 1981, Patt and colleagues found prolonged survival associated with inadvertent occlusion of hepatic artery [10, 11]. In 1983, Yamada and associates reported transarterial embolization (TAE) in 120 cases of patients with unresectable hepatoma [12], and Konno combined HAIC and TAE with ethiodol together, named TACE for hepatoma [13]. Since then, both TAE and TACE were widely applied with different embolization agents and chemical drugs, but their effects were controversial [14–19] until two randomized controlled trials (RCT) with high quality in 2002 [3, 4] and one systematic review in 2003 approved the benefits of TACE [20]. After that, TACE became the first line therapy for patients with intermediate stage of hepatoma [2]. Since no RCT with high quality compared TAE with best supportive care, TAE was not as widely recommended by guidelines as TACE [21] and only applied for a minority of appropriate patients in real world. In fact, TAE achieved similar, if not superior, results for patients with unresectable HCC compared with TACE in several RCTs [4, 22–26]. In this systematic review, we will summarize the results of all RCTs comparing TAE vs. TACE for patients with unresectable HCC.

## Methods

### Criteria for considering studies for this review

Inclusion criteria: (i) Study design: only randomized controlled trials (RCT) were considered; (ii) Study population: >18 years old, without gender restrictions, diagnosed with unresectable HCC regardless of etiology; (iii) Therapy for HCC: transarterial chemoembolization compared with embolization alone regardless of types of embolization agents and chemical drugs; (iv) Results available on progression-free survival (PFS) or overall survival (OS). Exclusion criteria: Primary HCC was treated with radical therapy (resection, ablation or transplantation), systemic therapy (chemotherapy, targeted therapy or immune therapy) or other local reginal therapy (internal or external radiotherapy).

### Search methods for identification of studies

We performed a systematic search of electronic databases (PubMed, EMBASE, Science Citation Index Expanded and Cochrane Library databases) for studies without language restriction (last literature search date: April 30, 2024). The search strategy was based on MeSH terms combined with free text words. The search strategies were given in Supplementary Table S1 and similar to our published work [27]. Reference lists of associated papers (included studies and relevant reviews) were checked as hand searching.

### Data collection and assessment of bias

Studies was screened according to the inclusion and exclusion criteria and data was extracted through a predesigned data extraction form by two authors independently. For duplicated publications, all the data was included but duplicated data was discarded if technically feasible but they were considered as one study. All included studies were assessed for methodological quality by two authors independently, as recommended by the Cochrane Hand book for RCTS [28]. Any disagreement between the two authors was resolved through discussion. OS was primary outcome. PFS, objective response rate (ORR) and adverse effects were secondary outcomes. We would contact and request the researchers to provide key missed information.

### Statistical analysis

We performed this systematic review according to the Cochrane Handbook [28] and reported in line with the Preferred reporting items for systematic reviews and meta-analyses (PRISMA) statement [29]. Hazard ratio (HR) between two arms was applied as a summary statistic for time-to-event outcomes like PFS and OS whereas Odds ratio (OR) was applied for dichotomous outcomes. HR and its standard error of each trial was calculated by a method described by Tierney and colleagues [30].

HR/OR of individual trials were pooled into an overall HR/OR by random-effects model. In accordance with customary, an overall HR/OR < 1 favored the TACE group and the difference was considered statistically significant if the 95% CI of the HR/OR didn’t overlap 1. Funnel plots would be used to evaluate the publication bias if there were sufficient studies. Sensitivity analyses were used to evaluate the reliability of the results.

Two authors input the data into RevMan 5.4.1 (Cochrane Collaboration, Oxford, UK), and performed all the analysis independently.

## Results

### Description of studies

**Fig. 1:**
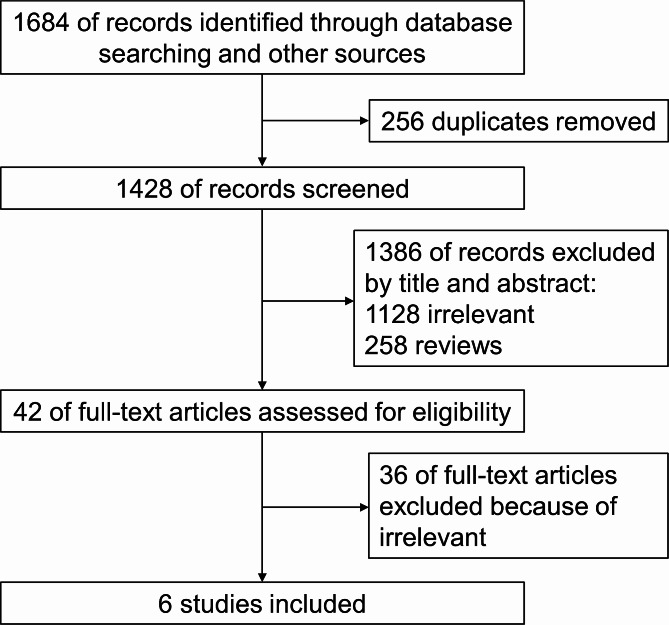
Study flow diagram

**Fig. 2:**
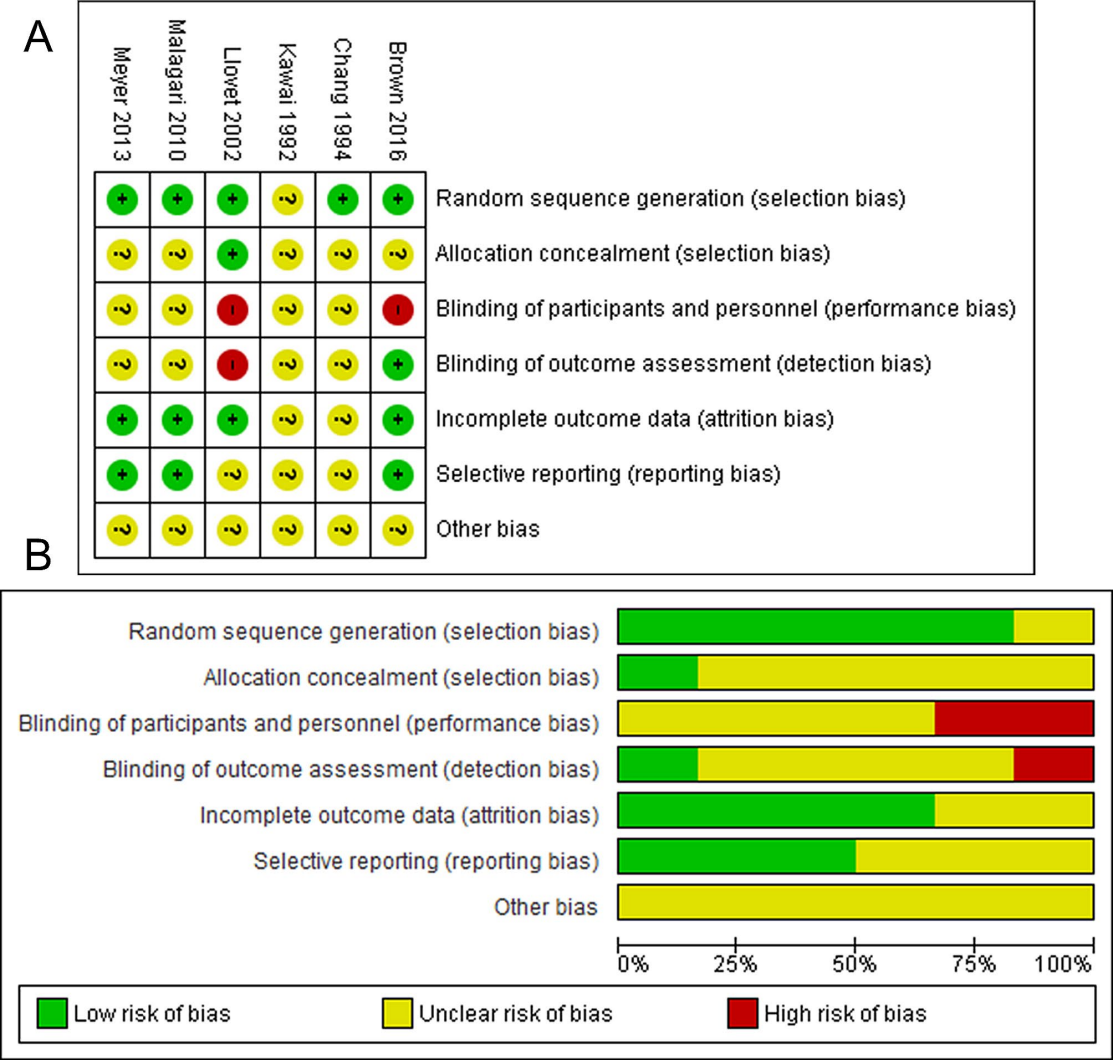
Risk of bias. **(A)** Risk of bias summary: review authors’ judgements about each risk of bias item for each included study; **(B)** Risk of bias graph: review authors’ judgements about each risk of bias item presented as percentages across all included studies

The study screening process is shown in Fig. 1. Six RCTs were included for this systematic review (Table 1) [4, 22–26]. Thirty six studies were excluded because they did not meet the inclusion criteria (Supplementary Table S2), including one just analyzed the data of patients who subsequently underwent liver resection (31/99, 31.3%) in each group after randomization [14], and another was not a randomized trial [31]. Three studies applied lipiodol or gelatin sponge [4, 22, 23] for embolization whereas others applied different kinds of microspheres [24–26]. Four RCTs used doxorubicin as chemical drugs for TACE [4, 22, 24, 26] and two studies used cisplatin [23, 25]. A total of 683 patients were included in this systematic review, among which 345 were in TACE-group whereas 338 in TAE-group. Child-Pugh Score was mainly A to B, and ECOG-PS (Eastern Cooperative Oncology Group-performance status) Score was mainly 0 to 1. But patients with tumors of BCLC (Barcelona Clinic Liver Cancer) stage A/B/C and etiology of HBV/HCV/alcohol were all included. The mean embolization number was from 1.37 to 3.1 for one patient (Table 1). The risk of bias of included RCT was from unclear to high risk (Fig. 2).

**Table 1:** Characteristics of the included studies

	HBV/ HCV	Country	Sample size (CE/E)	Male (CE/E)	Mean age (year) (CE/E)	Child-Pugh class (A/B) (CE; E)	ECOG-PS (0/1) (CE; E)	BCLC stage (A/B/C) (CE; E)	treatment details	Mean procedure No. (CE/E)	follow up (months) (CE/E)
Kawai 1992	NA	Japan	148/141	125/118	61/62	107/33; 102/25	71/38; 77/36	NA	CE: lipiodol/Adriamycin/ GS; E: lipiodol/GS	1.37	≈ 38/37 (longest)
Chang 1994	NA	Taiwan, China	22/24	20/23	64.1/63.8	13/9; 17/7	NA	NA	CE: cisplatin/Lipiodol/GS; E: Lipiodol/GS	2.8/3	≈ 26/27 (longest)
Llovet 2002	HBV8% HCV82%	Spain	40/37	32/30	63/64	31/9; 27/10	35/4; 28/7	0/35/5; 0/28/9	CE: doxorubicin/lipiodol/ GS; E: GS	2.8/3.1	21·2/21·7 (mean)
Malagari 2010	NA	Greece	41/43	31/34	70.7/70	NA	26/15; 28/15	23/18/0; 26/17/0	CE: doxorubicin/DC Beads; E: DC Beads	1–3	≈ 12/12 (longest)
Meyer 2013	HBV16% HCV41%	United Kindom	44/42	39/35	63.2/62.6	38/6; 33/9	31/8; 27/9	11/18/12; 9/16/15	CE: cisplatin/PVA; E: PVA	1–3	24/24 (median)
Brown 2016	HBV15% HCV30%	United States	50/51	41/37	65.5/68.3	45/5; 41/10	43/7; 44/7	12/23/15; 10/22/19	CE: doxorubicin/LCB/ PVA; E: BB/PVA;	2/2	34/34 (median)

**Abbreviations**: CE: short for TACE (transarterial chemoembolization); E: short for TAE (transarterial embolization); ECOG PS: Eastern Cooperative Oncology Group performance status; BCLC: Barcelona Clinic Liver Cancer; No.: number; NA: not available; GS: gelatin sponge; DC Beads: a kind of drug eluting bead from Terumo (Biocompatibles); PVA: polyvinyl alcohol; LCB: LC Bead (Biocompatibles UK), can be loaded with doxorubicin; BB: Bead Block (Biocompatibles UK, Farnham, Surrey, United Kingdom); HBV: hepatitis B virus; HCV: hepatitis C virus;

### Effects of intervention

**Fig. 3:**
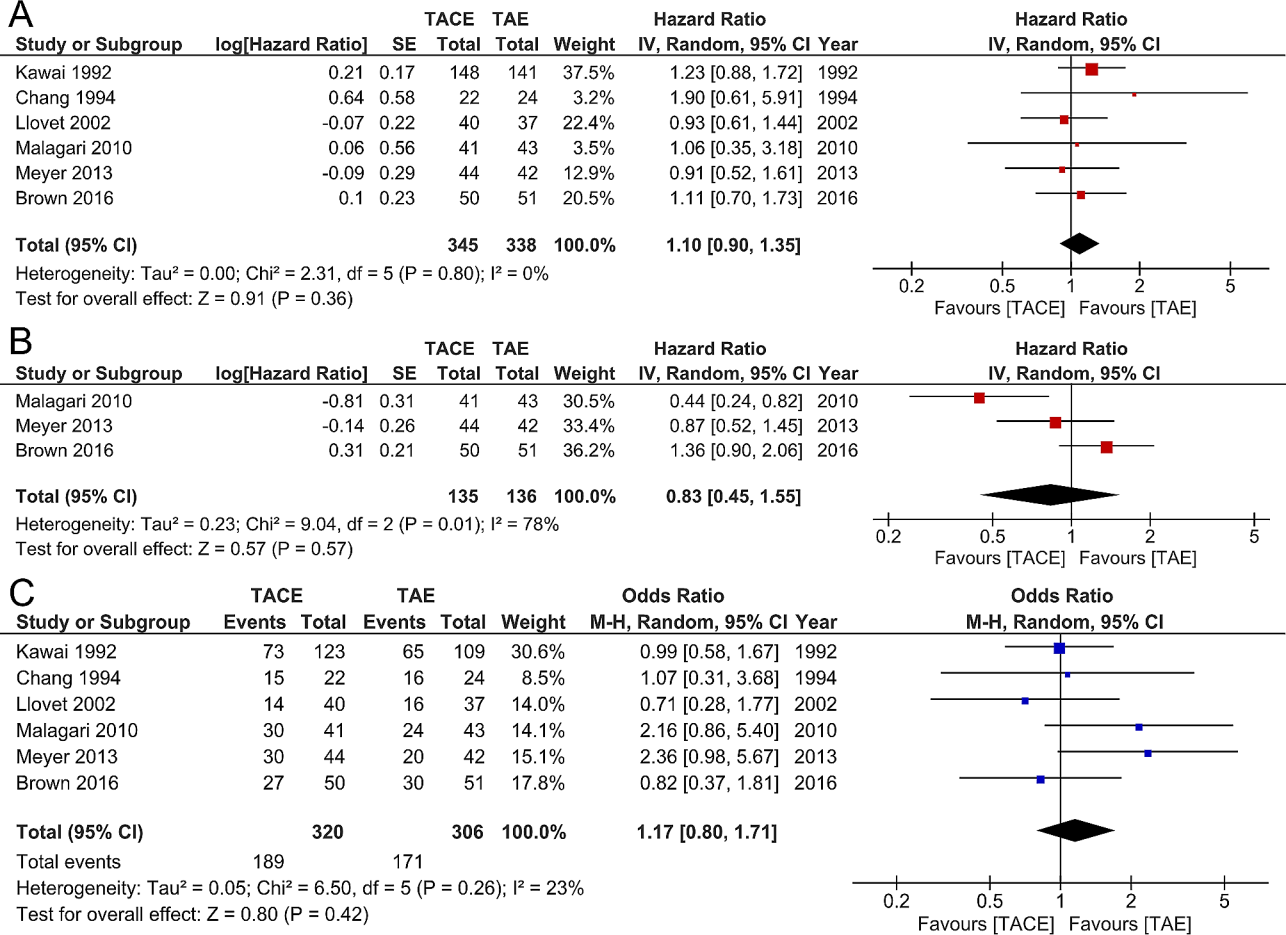
Forest plot of 6 studies comparing TACE with TAE for unresectable HCC. Forest plot showing no significant difference between TACE and TAE for overall survival **(A)**, progression-free survival **(B)** and objective response rate **(C)**

**Fig. 4:**
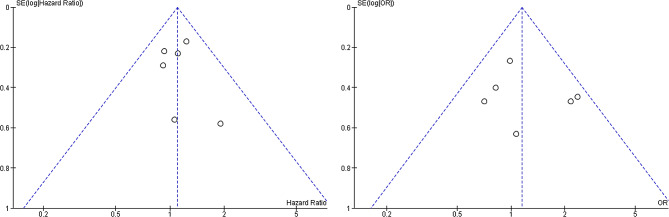
Funnel plot for assessing publication bias. Funnel plot showing symmetry indicative no obvious publication bias for overall survival **(A)** and objective response rate **(B)**

Pooling the data of six studies [4, 22–26] that assessed OS in 683 patients showed no significant difference between TACE and TAE (HR 1.10, 95% CI 0.90–1.35; *p* = 0.36), without significant between-study heterogeneity [X^2^ = 2.31, degrees of freedom (df) = 5; *p* = 0.80; I^2^ = 0%] (Fig. 3A). All the six studies reported ORR and pooled data showed no significant difference between TACE and TAE (OR 1.17, 95% CI 0.80–1.71; *p* = 0.42), without significant between-study heterogeneity [X^2^ = 6.50, df = 5; *p* = 0.26; I^2^ = 23%] (Fig. 3C). No significant publication bias was found by funnel plots (Fig. 4) for both OS and ORR. Only three studies reported PFS and pooled data showed no significant difference between two groups (HR 0.83, 95% CI 0.45–1.55; *p* = 0.57), with significant between-study heterogeneity [X^2^ = 9.04, df = 2; *p* = 0.01; I^2^ = 78%] (Fig. 3B). Funnel plot was not shown due to insufficient studies.

### Sensitivity analysis

Sensitivity analyses of studies excluding studies with high risk of bias, or studies applied lipiodol or gelatin sponge as embolization materials, or studies applied microsphere as embolization materials (all of them were published after 2010), or studies applied doxorubicin as chemical drugs, still showed no significant difference between the TACE and TAE group (Table 2).

**Table 2:** Sensitivity analyses comparing TACE versus TAE

	No. of studies	No. of patients			HR/OR (95% CI)	*p*-value	Study heterogeneity			
		TACE	TAE	total			X^2^	df	I^2^	*p*-value
**Excluding studies with high risk of bias**										
OS	4	255	250	505	1.17 [0.89, 1.53]	0.25	1.55	3	0%	0.67
ORR	4	230	218	448	1.35 [0.93, 1.98]	0.12	4.05	3	26%	0.26
**Studies applied lipiodol or gelatin sponge as embolization materials**										
OS	3	210	202	412	1.14 [0.88, 1.48]	0.31	1.82	2	0%	0.40
ORR	3	185	170	355	0.93 [0.61, 1.42]	0.73	0.44	2	0%	0.80
**Studies applied microsphere as embolization materials (all of them were published after 2010)**										
OS	3	135	136	271	1.03 [0.74, 1.44]	0.86	0.27	2	0%	0.88
ORR	3	135	136	271	1.52 [0.93, 2.47]	0.09	3.87	2	48%	0.14
**Studies applied doxorubicin as chemical drugs**										
OS	4	279	272	551	1.11 [0.89, 1.38]	0.37	1.02	3	0%	0.80
ORR	4	254	240	494	1.02 [0.71, 1.47]	0.90	3.49	3	14%	0.32

**Abbreviation**: No., number; TACE: transarterial chemoembolization; TAE: transarterial embolization; HR: hazard ratio; OR: odds ratio; CI: confidence interval; df: degrees of freedom; OS: overall survival; ORR: objective response rate

### Adverse effects

Meta-analysis comparing adverse effects could not be achieved due to lack of consistency in reporting. Kawai 1992 reported significant lower hemoglobin level in TACE group than TAE group whereas all the other blood cells and liver function, as well as abdominal pain and fever were similar in two groups [22]. Chang 1994 reported higher incidence of emesis in TACE group (75.4% vs. 9.6%) due to cisplatin whereas all the others like liver function and renal function were similar [23]. In Llovet 2002, one patient died due to septic shock in TACE group and TACE group also had higher number of treatment-related complications (11 vs. 7) [4]. Both postembolization syndrome and other complications were similar in Malagari 2010 and Brown 2016 [24, 26]. Meyer 2013 reported higher grade 3/4 adverse events in TACE group (83.7% vs. 60.5%) but similar quality of life between the two groups [25]. In summary, TAE group reported similar or less adverse effects than TACE group in all the studies. The detailed adverse events were shown in Supplementary Table S3.

## Discussion

In this systematic review, six RCTs fulfilled our criteria. The risk of bias of included RCTs was from unclear to high risk. All the studies applied more than 1 procedure for each patient if possible or needed. The results showed that TAE was as effective as TACE for both PFS and OS, as well as ORR. Sensitivity analysis showed similar results in two groups regardless of embolization agents or chemical drugs or excluded studies with high risk of bias. But TAE group reported similar or less adverse effects than TACE group. Three of the six studies used lipiodol or gelatin sponge, alone or together as embolization agents [4, 22, 23], but for other three studies, one applied polyvinyl alcohol (PVA) microsphere [25] and the other two studies applied drug-eluting beads [24, 26]. Four studies applied doxorubicin whereas two studies employed cisplatin [23, 25]. Our results were similar to three published systematic reviews, which pooled published data and found no significant difference in mortality between the two groups [25, 32, 33]. But two [25, 32] of them did not apply HR as a summary statistic for time-to-event outcome like OS. And one most recent review [33] did not include one important RCT published in the Lancet [4], which we think totally fulfilled the inclusion criteria. We did not find any other published systematic review and meta-analysis focusing on this issue.

Since hepatic arterial infusion chemotherapy (HAIC) was introduced into clinic much earlier than TAE [9, 12], and its effects were also been fully improved and widely accepted [11, 34]. Undoubtedly, no one doubts the value of retaining chemotherapy during TAE. However, only continuous infusion of chemotherapy with small dose had obvious anti-cancer effects, which has been proved by two recent RCTs [35, 36]. In their studies, HAIC with FOLFOX (Oxaliplatin, Calcium folinate and 5-Fluorouracil) regimen even improved overall survival than TACE or sorafenib for patients with unresectable HCC. During the procedure of TACE, chemical drugs were injected into hepatic artery transiently. Though it’s widely believed that after the emulsion of anti-cancer drugs with lipiodol, the drugs can deposit into tumor tissue with lipiodol for a long time. No one can tell how many and how long the drugs deposited in situ. Not to mention the heterogeneity of tumors, the individual differences and technical inconsistency [37, 38]. According to our study and others TACE was not superior to TAE and HAIC, we speculated that either due to doxorubicin/cisplatin was not as effective as FOLFOX for HCC, or only a few drugs deposited into the tumor tissue. Another concern is about the extent of embolization. Traditional embolization aims to embolize as much tumor tissue as possible, which will leads to damage to liver function, even after introduction of super-selective embolization. This kind of strategy will limit the upper-amount numbers of procedure as well as the probability of successful conversion as hepatectomy requires adequate liver reserve. In order to overcome this dilemma, the combination of embolization of collateral feeding arteries with HAIC of main feeding artery should be a good strategy (TAE + HAIC) [39].

Some limitations of this study should be discussed. First of all, all included RCTs had unclear or high risk of bias. But the results were stable in between studies and according to sensitivity analysis. Second, significant between-study heterogeneity existed because of the different patients (etiology, characteristics of tumors, et al.), types of embolization agents and anti-cancer drugs as well as their doses, number of procedures for each patient as well as interval between each procedure. Though we applied random-effects model where appropriate and sensitivity analysis, but their credibility was decreased by relatively small number of included studies. Third, the definitions of outcomes were not the same in between studies since the earliest included study being published in 1992 and the latest published in 2016 (Supplementary Table S4). Fourth, only six RCTs fulfilled our including criteria. The sample size was not big enough. Fifth, PFS was not available for half of studies and pooling the data of adverse effects was not achieved despite the efforts made to contact the authors. Sixth, though we are confident the un-inferiority of TAE compared to TACE for unresectable HCC, but the exact reason is still not clear.

## Conclusions

Despite these limitations listed above, our study still demonstrated that the anti-cancer effects of TAE were at least as well as, if not superior than TACE. Since TAE was simpler, cheaper and had less adverse effects than TACE, TAE should be a better choice in most cases where TACE was indicated for unresectable HCC. Recent studies demonstrated the advantage of HAIC than TACE, further studies should be focused on the combination of TAE and HAIC even systematic therapy in order to offset their respective disadvantages and achieve the best effect [40, 41].

### Electronic supplementary material

Below is the link to the electronic supplementary material.

Supplementary Material 1

Supplementary Material 2

Supplementary Material 3

Supplementary Material 4

## Data Availability

Data sharing is not applicable to this article as no new data were created or analyzed.

## Data availability

All data used within this study have been publicly available. The corresponding author will consider sharing the dataset gathered upon request.

## Abbreviations

TACE: transarterial chemoembolization
HCC: hepatocellular carcinoma
TAE: transarterial embolization
RCTs: randomized controlled studies
HR: Hazard Ratio
OR: Odds Ratio
HAIC: Hepatic arterial infusion chemotherapy
PFS: progression-free survival
OS: overall survival
ORR: objective response rate
ECOG-PS: Eastern Cooperative Oncology Group-performance status
BCLC: Barcelona Clinic Liver Cancer
FOLFOX: Oxaliplatin, Calcium folinate and 5-Fluorouracil
