# Supplementary material for: Embolization alone is as effective as TACE for unresectable HCC: systematic review and meta-analysis of randomized controlled trails
Source: BMC Gastroenterol. 2024 Jun 7;24:195. doi: 10.1186/s12876-024-03282-z (PMC11162027; doi:10.1186/s12876-024-03282-z)
Supplement: Supplementary file 1 — Supplementary Material 1 [file 12876_2024_3282_MOESM1_ESM.doc]

**Supplementary Table S2.** Excluded studies

|  | **Excluded reason** |
| --- | --- |
| **Konno 1983[1]** | The intervention was not TACE or TAE. |
| **Yamada 1983[2]** | It was not a RCT. |
| **Takayasu 1987[3]** | This study analyzed the data of patients who subsequently underwent liver resection (31/99, 31.3%) in each group after randomization. |
| **Kasugai 1989[4]** | It was not a RCT. |
| **Aoyama 1992[5]** | It was not a RCT. |
| **Okamura 1992[6]** | The intervention was TACE with different drugs. |
| **Kawai 1994[7]** | The intervention was TACE with different drugs. |
| **Motohara 1994[8]** | It was not a RCT. |
| **Hatanaka 1995[9]** | It was not a RCT. |
| **Kawai 1997[10]** | The intervention was TACE with different drugs. |
| **Kwok 2000[11]** | The intervention was TACE with different material for embolization. |
| **Trevisani 2001[12]** | It was a review. |
| **Camma 2002[13]** | It was a systematic review. |
| **Geschwind 2002[14]** | It was a review. |
| **Llovet 2003[15]** | It was a systematic review. |
| **Ikeda 2004[16]** | The intervention of one group was transcatheter arterial chemotherapy without embolization. |
| **Reidy 2004[17]** | It was a review. |
| **Marelli 2007[18]** | It was a review. |
| **Lu 2008[19]** | The intervention was TACE with drug of different doses. |
| **Okusaka 2009[20]** | The intervention of one group was transcatheter arterial chemotherapy without embolization. |
| **Boulin 2011[21]** | The intervention was TACE with different drugs. |
| **Carr 2011[22]** | The intervention of one group was transcatheter arterial chemotherapy without embolization. |
| **Oliveri 2011[23]** | It was a systematic review. |
| **Salhab 2011[24]** | It was a systematic review. |
| **Yamasaki 2011[25]** | The intervention was TACE with different material for embolization. |
| **Brown 2012[26]** | It was a review. |
| **Takayasu 2012[27]** | It was a review. |
| **Chan 2013[28]** | The intervention was TACE with different material for embolization. |
| **Shi 2013[29]** | The intervention was TACE with different material for embolization. |
| **Lencioni 2016[30]** | It was a review. |
| **Facciorusso 2017[31]** | It was a systematic review. |
| **Ikeda 2018[32]** | The intervention was TACE with different drugs. |
| **Wang 2018[33]** | It was not a RCT. |
| **He 2018[34]** | The intervention was TACE with different material for embolization. |
| **Swierz 2020[35]** | There was no embolization in one group. |
| **Haber 2021[36]** | It was a systematic review. |

**Abbreviations:** TACE: transarterial chemoembolization; TAE: transarterial embolization; RCT: randomized controlled trial.

**Reference**

1. Konno, T., et al., *Effect of arterial administration of high-molecular-weight anticancer agent SMANCS with lipid lymphographic agent on hepatoma: a preliminary report.* Eur J Cancer Clin Oncol, 1983. **19**(8): p. 1053-65.

2. Yamada, R., et al., *Hepatic artery embolization in 120 patients with unresectable hepatoma.* Radiology, 1983. **148**(2): p. 397-401.

3. Takayasu, K., et al., *Hepatocellular carcinoma: treatment with intraarterial iodized oil with and without chemotherapeutic agents.* Radiology, 1987. **163**(2): p. 345-51.

4. Kasugai, H., et al., *Treatment of hepatocellular carcinoma by transcatheter arterial embolization combined with intraarterial infusion of a mixture of cisplatin and ethiodized oil.* Gastroenterology, 1989. **97**(4): p. 965-71.

5. Aoyama, K., et al., *Evaluation of transcatheter arterial embolization with epirubicin-lipiodol emulsion for hepatocellular carcinoma.* Cancer Chemother Pharmacol, 1992. **31 Suppl**: p. S55-9.

6. Okamura, J., et al., *Prospective and randomized clinical trial for the treatment of hepatocellular carcinoma--a comparison of L-TAE with Farmorubicin and L-TAE with adriamycin (second cooperative study). The Cooperative Study Group for Liver Cancer Treatment of Japan.* Cancer Chemother Pharmacol, 1992. **31 Suppl**: p. S20-4.

7. Kawai, S., et al., *Prospective and randomized clinical trial for the treatment of hepatocellular carcinoma--a comparison between L-TAE with farmorubicin and L-TAE with adriamycin: preliminary results (second cooperative study). Cooperative Study Group for Liver Cancer Treatment of Japan.* Cancer Chemother Pharmacol, 1994. **33 Suppl**: p. S97-102.

8. Motohara, T., Z. Ozawa, and S. Morita, *[Intra-arterial chemotherapy for the treatment of advanced hepatocellular carcinoma through implantable port (reservoir)].* Gan To Kagaku Ryoho, 1994. **21**(15): p. 2645-8.

9. Hatanaka, Y., et al., *Unresectable hepatocellular carcinoma: analysis of prognostic factors in transcatheter management.* Radiology, 1995. **195**(3): p. 747-52.

10. Kawai, S., et al., *Prospective and randomized trial of lipiodol-transcatheter arterial chemoembolization for treatment of hepatocellular carcinoma: a comparison of epirubicin and doxorubicin (second cooperative study). The Cooperative Study Group for Liver Cancer Treatment of Japan.* Semin Oncol, 1997. **24**(2 Suppl 6): p. S6-38-S6-45.

11. Kwok, P.C., et al., *A randomized clinical trial comparing autologous blood clot and gelfoam in transarterial chemoembolization for inoperable hepatocellular carcinoma.* J Hepatol, 2000. **32**(6): p. 955-64.

12. Trevisani, F., et al., *Randomized control trials on chemoembolization for hepatocellular carcinoma: is there room for new studies?* J Clin Gastroenterol, 2001. **32**(5): p. 383-9.

13. Camma, C., et al., *Transarterial chemoembolization for unresectable hepatocellular carcinoma: meta-analysis of randomized controlled trials.* Radiology, 2002. **224**(1): p. 47-54.

14. Geschwind, J.F., *Chemoembolization for hepatocellular carcinoma: where does the truth lie?* J Vasc Interv Radiol, 2002. **13**(10): p. 991-4.

15. Llovet, J.M. and J. Bruix, *Systematic review of randomized trials for unresectable hepatocellular carcinoma: Chemoembolization improves survival.* Hepatology, 2003. **37**(2): p. 429-42.

16. Ikeda, M., et al., *Transcatheter arterial chemotherapy with and without embolization in patients with hepatocellular carcinoma.* Oncology, 2004. **66**(1): p. 24-31.

17. Reidy, D.L. and J.D. Schwartz, *Therapy for unresectable hepatocellular carcinoma: review of the randomized clinical trials-I: hepatic arterial embolization and embolization-based therapies in unresectable hepatocellular carcinoma.* Anticancer Drugs, 2004. **15**(5): p. 427-37.

18. Marelli, L., et al., *Transarterial therapy for hepatocellular carcinoma: which technique is more effective? A systematic review of cohort and randomized studies.* Cardiovasc Intervent Radiol, 2007. **30**(1): p. 6-25.

19. Lu, W., et al., *Necrosis and apoptosis in hepatocellular carcinoma following low-dose versus high-dose preoperative chemoembolization.* Cardiovasc Intervent Radiol, 2008. **31**(6): p. 1133-40.

20. Okusaka, T., et al., *Transarterial chemotherapy alone versus transarterial chemoembolization for hepatocellular carcinoma: a randomized phase III trial.* J Hepatol, 2009. **51**(6): p. 1030-6.

21. Boulin, M., et al., *Randomised controlled trial of lipiodol transarterial chemoembolisation with or without amiodarone for unresectable hepatocellular carcinoma.* Dig Liver Dis, 2011. **43**(11): p. 905-11.

22. Carr, B.I., K. Bron, and D.P. Swanson, *Prospective randomized trial of hepatic artery chemotherapy with cisplatin and doxorubicin, with or without lipiodol in the treatment of advanced stage hepatocellular carcinoma.* J Clin Gastroenterol, 2011. **45**(9): p. e87-91.

23. Oliveri, R.S., J. Wetterslev, and C. Gluud, *Transarterial (chemo)embolisation for unresectable hepatocellular carcinoma.* Cochrane Database Syst Rev, 2011(3): p. Cd004787.

24. Salhab, M. and R. Canelo, *An overview of evidence-based management of hepatocellular carcinoma: a meta-analysis.* J Cancer Res Ther, 2011. **7**(4): p. 463-75.

25. Yamasaki, T., et al., *A novel transcatheter arterial infusion chemotherapy using iodized oil and degradable starch microspheres for hepatocellular carcinoma: a prospective randomized trial.* J Gastroenterol, 2011. **46**(3): p. 359-66.

26. Brown, D.B., et al., *Quality improvement guidelines for transhepatic arterial chemoembolization, embolization, and chemotherapeutic infusion for hepatic malignancy.* J Vasc Interv Radiol, 2012. **23**(3): p. 287-94.

27. Takayasu, K., *Transarterial chemoembolization for hepatocellular carcinoma over three decades: current progress and perspective.* Jpn J Clin Oncol, 2012. **42**(4): p. 247-55.

28. Chan, S.L., et al., *Re: roles played by chemolipiodolization and embolization in chemoembolization for hepatocellular carcinoma: single-blind, randomized trial.* J Natl Cancer Inst, 2013. **105**(8): p. 580.

29. Shi, M., et al., *Roles played by chemolipiodolization and embolization in chemoembolization for hepatocellular carcinoma: single-blind, randomized trial.* J Natl Cancer Inst, 2013. **105**(1): p. 59-68.

30. Lencioni, R., et al., *Lipiodol transarterial chemoembolization for hepatocellular carcinoma: A systematic review of efficacy and safety data.* Hepatology, 2016. **64**(1): p. 106-16.

31. Facciorusso, A., et al., *Transarterial chemoembolization vs bland embolization in hepatocellular carcinoma: A meta-analysis of randomized trials.* United European Gastroenterol J, 2017. **5**(4): p. 511-518.

32. Ikeda, M., et al., *Transarterial chemoembolization with miriplatin vs. epirubicin for unresectable hepatocellular carcinoma: a phase III randomized trial.* J Gastroenterol, 2018. **53**(2): p. 281-290.

33. Wang, Y., et al., *[Transarterial chemoembolization with bleomycin treatment for moderate-advenced hepatocellular carcinoma].* Zhonghua Yi Xue Za Zhi, 2018. **98**(39): p. 3166-3170.

34. He, M.K., et al., *Comparison of Stable and Unstable Ethiodized Oil Emulsions for Transarterial Chemoembolization of Hepatocellular Carcinoma: Results of a Single-Center Double-Blind Prospective Randomized Controlled Trial.* J Vasc Interv Radiol, 2018. **29**(8): p. 1068-1077.e2.

35. Swierz, M.J., et al., *Transarterial (chemo)embolisation versus no intervention or placebo for liver metastases.* Cochrane Database Syst Rev, 2020. **3**(3): p. Cd009498.

36. Haber, P.K., et al., *Evidence-Based Management of Hepatocellular Carcinoma: Systematic Review and Meta-analysis of Randomized Controlled Trials (2002-2020).* Gastroenterology, 2021. **161**(3): p. 879-898.
