# Supplementary material for: Embolization alone is as effective as TACE for unresectable HCC: systematic review and meta-analysis of randomized controlled trails
Source: BMC Gastroenterol. 2024 Jun 7;24:195. doi: 10.1186/s12876-024-03282-z (PMC11162027; doi:10.1186/s12876-024-03282-z)
Supplement: Supplementary file 2 — Supplementary Material 2 [file 12876_2024_3282_MOESM2_ESM.docx]

**Supplementary Table S3.** Adverse events.

Kawai 1992

|  | TACE (134) | TAE (115) |
| --- | --- | --- |
| WBC decrease | 34 (25.4%) | 20 (17.4%) |
| Hb decrease | 30 (22.4%) | 11 (9.6%)^#^ |
| platelet decrease | 19 (14.2%) | 22 (19.1%) |
| abdominal pain | 87 (64.9%) | 74 (64.3%) |
| fever | 108 (80.6%) | 92 (80.0%) |
| liver function | No difference | |

Chang 1994

|  | TACE (22) | TAE (24) |
| --- | --- | --- |
| emesis | 75.4% | 9.6%^#^ |
| major complications | 2 (9.1%) | 1 (4.2%) |
| renal function | No difference | |
| liver function | No difference | |

Llovet 2002

|  | TACE (40) | TAE (37) |
| --- | --- | --- |
| major complications | 11 (27.5%) | 7 (18.9%) |
| death | 1 (2.5%) | 0 |

Malagari 2010

|  | TACE (41) | TAE (43) |
| --- | --- | --- |
| postembolization syndrome | 33 (80.4%) | 35 (81.4%) |
| liver function | No difference | |
| major complications | 11 (26.8%) | 7 (18%) |
| death | 0 | 0 |

Meyer 2013 (44/42)

|  | TACE (44) | TAE (42) |
| --- | --- | --- |
| myelosuppression (grade 3) | 8 (18.2%) | 0^#^ |
| myelosuppression (grade 4) | 0 | 0 |
| liver function (grade 3) | 32 (72.7%) | 19 (45.2%)^#^ |
| liver function (grade 4) | 3 (6.8%) | 3 (7.1%) |
| constitutional AEs (grade 3) | 26 (59.1%) | 22 (52.4%) |
| constitutional AEs (grade 4) | 7 (15.9%) | 4 (9.5%) |
| overall grade 3/4 | 83.7% | 63.5%^#^ |
| death | 1 (2.3%) | 1 (2.4%) |
| quality of life | No difference | |

Brown 2016 (50/51)

|  | TACE (50) | TAE (51) |
| --- | --- | --- |
| postembolization syndrome | 84% | 88% |
| grade 3 | 49 (98%) | 41 (80.4%)^#^ |
| grade 4 | 21 (42%) | 19 (37.3%) |
| major complications | 6 (12%) | 6 (11.7%) |
| total SAEs | 71 | 61 |
| number or grade of SAE | No difference | |
| number of SAEs per patient | No difference | |
| highest grade of SAE | No difference | |
| death | 1 (2%) | 1 (2%) |

**Abbreviations:** TACE: transarterial chemoembolization; TAE: transarterial embolization; WBC: white blood cells; Hb: hemoglobin; AE: adverse events; SAE: serious adverse events; ^#^: Significant difference between two groups.
