# Supplementary material for: Embolization alone is as effective as TACE for unresectable HCC: systematic review and meta-analysis of randomized controlled trails
Source: BMC Gastroenterol. 2024 Jun 7;24:195. doi: 10.1186/s12876-024-03282-z (PMC11162027; doi:10.1186/s12876-024-03282-z)
Supplement: Supplementary file 3 — Supplementary Material 3 [file 12876_2024_3282_MOESM3_ESM.docx]

**Supplementary Table S4.** Definitions of outcomes.

|  | CR/PR/SD/PD | complications |
| --- | --- | --- |
| Kawai 1992 | A decrease of tumor size > 25% in the two-dimensional area of the tumor was assessed to be responsive to the treatment. Or else, no response. | NA |
| Chang 1994 | If Lipiodol retention was > 50% in the treated tumor measured by post-TAE CT scan and/or angiography, the tumor was assessed to be responsive to the treatment, and if < 50%, it was considered stationary or progressive disease. | NA |
| Llovet 2002 | WHO criteria:  CR: no evidence of neoplastic disease; PR: reduction in total tumor load of more than 50%; SD: reduction of less than 50% or increase of less than 25%; PD: increase of equal to or more than 25%. Thus, objective responses accounted for CR and PR sustained for at least 6 months. | NA |
| Malagari 2010 | EASL classification:  CR: complete response; PR: decrease>50%; SD: decrease≤50% or increase≤25%; PD: local recurrence, increase>25% or new lesions. | NA |
| Meyer 2013 | RECIST 1.0 and mRECIST:  CR: no evidence of neoplastic disease; PR: > 30% decrease; SD: Neither PR nor PD criteria met; PD: 20% increase; | CTCAE 3.0 |
| Brown 2016 | RECIST 1.0 | CTCAE 3.0 |

**Abbreviations:**

CR: complete response; PR: partial response; SD: stable disease; PD: progressive disease; ORR: objective response rate=CR+PR; RECIST: response evaluation criteria in solid tumors; NA: not available; CTCAE: Common Terminology Criteria for Adverse Events
