# Supplementary material for: Embolization alone is as effective as TACE for unresectable HCC: systematic review and meta-analysis of randomized controlled trails
Source: BMC Gastroenterol. 2024 Jun 7;24:195. doi: 10.1186/s12876-024-03282-z (PMC11162027; doi:10.1186/s12876-024-03282-z)
Supplement: Supplementary file 4 — Supplementary Material 4 [file 12876_2024_3282_MOESM4_ESM.doc]

**Supplementary Table S1.** Search strategy

| **MEDLINE (PubMed),** from dates of inceptions up to April 30, 2024 |
| --- |
| #1 "Carcinoma, Hepatocellular"[Mesh]  #2 ((liver* OR hepatic OR hepatocellular* OR hepato-cellular) AND (carcinom* OR cancer* OR malign* OR tumo*)) OR HCC OR hepatoma*  #3 #1 OR #2  #4 "Embolization, Therapeutic"[Mesh] OR "Embolotherap*" [Supplementary Concept] OR "Embolizations, Therapeutic" [Supplementary Concept] OR "Therapeutic Embolizations" [Supplementary Concept]  #5 transarterial chemoembolization OR TACE OR transarterial embolization OR TAE  #6 #4 OR #5  #7 survival OR death OR mortalit* OR fatality OR recurrence* OR relapse* OR recrudescence*  #8 ((randomized controlled trial[pt]) OR (controlled clinical trial[pt]) OR (randomized[tiab]) OR (placebo[tiab]) OR (drug therapy[sh]) OR (randomly[tiab]) OR (trial[tiab]) OR (groups[tiab])) NOT (animals[mh] NOT humans[mh])  #9 #3 AND #6 AND #7 AND #8 |
| **EMBASE,** from dates of inceptions up to April 30, 2024 |
| #1 'liver cell carcinoma'/exp  #2 (liver* OR hepatic OR hepatocellular* OR 'hepato cellular') AND (carcinom* OR cancer* OR malign* OR tumo*) OR hcc OR hepatoma*  #3 #1 or #2  #4 'Embolization'/exp OR Embolotherap* OR Embolization*  #5 'transarterial chemoembolization' OR TACE OR 'transarterial embolization' OR TAE  #6 #4 OR #5  #7 survival OR death OR mortalit* OR fatality OR recurrence* OR relapse* OR recrudescence*  #8 random* OR blind* OR placebo OR 'meta analysis'  #9 #3 AND #6 AND #7 AND #8 |
| **Cochrane Library databases,** from dates of inceptions up to April 30, 2024 |
| #1 MeSH descriptor: [Carcinoma, Hepatocellular] explode all trees  #2 (((liver* or hepatic or hepatocellular* or hepato-cellular) and (carcinom* or cancer* or malign* or tumo*)) or HCC or hepatoma*)  #3 #1 or #2  #4 MeSH descriptor: [Embolization, Therapeutic] explode all trees  #5 ((transarterial and (embolization or chemoembolization)) or TACE or TAE or embolization* or Embolotherap*  #6 #4 OR #5  #7 #3 and #6  in Cochrane Reviews (Reviews only), Other Reviews and Trials (Word variations have been searched) |
| **Science Citation Index Expanded,** from dates of inceptions up to April 30, 2024 |
| #1 TS=(((liver* OR hepatic OR hepatocellular* OR hepato-cellular) AND (carcinom* OR cancer* OR neoplasm* OR malign* OR tumo*)) OR HCC OR hepatoma*)  #2 TS=( Embolotherap* OR embolization* OR chemoembolization* OR TACE OR TAE)  #3 TS=(survival OR death OR mortalit* OR fatality OR recurrence* OR relapse* OR recrudescence*)  #4 #3 AND #2 AND #1 |
